# Meningococcal Meningitis Surveillance in the African Meningitis Belt, 2004–2013

**DOI:** 10.1093/cid/civ597

**Published:** 2015-11-09

**Authors:** Clément Lingani, Cassi Bergeron-Caron, James M. Stuart, Katya Fernandez, Mamoudou H. Djingarey, Olivier Ronveaux, Johannes C. Schnitzler, William A. Perea

**Affiliations:** 1 Inter-country Support Team for West Africa, World Health Organization, Ouagadougou, Burkina Faso; 2 Université de Montréal, Canada; 3 Faculty of Infectious and Tropical Diseases, London School of Hygiene and Tropical Medicine, United Kingdom; 4 Department of Pandemic and Epidemic Diseases, World Health Organization, Geneva, Switzerland

**Keywords:** meningitis, Africa, surveillance

## Abstract

***Background.*** An enhanced meningitis surveillance network was established across the meningitis belt of sub-Saharan Africa in 2003 to rapidly collect, disseminate, and use district weekly data on meningitis incidence. Following 10 years’ experience with enhanced surveillance that included the introduction of a group A meningococcal conjugate vaccine, PsA-TT (MenAfriVac), in 2010, we analyzed the data on meningitis incidence and case fatality from countries reporting to the network.

***Methods.*** After de-duplication and reconciliation, data were extracted from the surveillance bulletins and the central database held by the World Health Organization Inter-country Support Team in Burkina Faso for countries reporting consistently from 2004 through 2013 (Benin, Burkina Faso, Chad, Democratic Republic of Congo, Ghana, Côte d'Ivoire, Mali, Niger, Nigeria, Togo).

***Results.*** The 10 study countries reported 341 562 suspected and confirmed cases over the 10-year study period, with a marked peak in 2009 due to a large epidemic of group A *Neisseria meningitidis* (NmA) meningitis. Case fatality was lowest (5.9%) during this year. A mean of 71 and 67 districts annually crossed the alert and epidemic thresholds, respectively. The incidence rate of NmA meningitis fell >10-fold, from 0.27 per 100 000 in 2004–2010 to 0.02 per 100 000 in 2011–2013 (*P* < .0001).

***Conclusions.*** In addition to supporting timely outbreak response, the enhanced meningitis surveillance system provides a global overview of the epidemiology of meningitis in the region, despite limitations in data quality and completeness. This study confirms a dramatic fall in NmA incidence after the introduction of PsA-TT.

Countries in the African “meningitis belt,” an area in sub-Saharan Africa that stretches from Senegal in the west to Ethiopia in the east, are susceptible to intermittent devastating outbreaks of meningococcal meningitis, with attack rates as high as 1% of the population during major epidemics [1]. Most epidemics have been due to group A *Neisseria meningitidis* (NmA), and some have been due to groups C, W, and X (NmC, NmW, and NmX, respectively). In 2003, countries put in place an enhanced meningitis surveillance network with support of the World Health Organization (WHO) and the Meningitis Vaccine Project, a joint WHO/PATH initiative [2]. Standard operating procedures for surveillance implementation were developed and disseminated to participating countries; in short, data on suspected cases and deaths are recorded and transmitted weekly from health centers to the district medical officer, including zero reporting, and onward by radio, telephone, fax, or email to provincial and national levels [3]. When available, cerebrospinal fluid (CSF) specimens are sent to microbiology laboratories for the identification of the pathogen by rapid diagnostic test, polymerase chain reaction (PCR), latex agglutination test, or culture. Each country reports to the WHO Inter-country Support Team (WHO/IST) for West Africa each week in the meningitis season, and monthly the rest of the year.

An electronic application was developed by WHO for the compilation and analysis of the data. The system was implemented in 2005, with the database hosted in WHO/IST. Following initial variation in the format of data on cases and deaths provided by week and district, WHO/IST worked with reporting countries to standardize the format. Most participating countries are now providing data on the number of cases and deaths by district and week in an electronic format based on a standardized Microsoft Excel format or Microsoft Access. All files are automatically extracted and compiled in one database. Automated analysis and processing of the data are carried out using the open-source software package R [4]. The automated output for the bulletin includes tables, charts, maps, calculation of districts in epidemic and alert, with the addition of customized maps and an assessment of the current situation by WHO/IST [5].

Initially, in 2003, 8 countries (Benin, Burkina Faso, Chad, Ghana, Mali, Niger, Nigeria, and Togo) contributed to this enhanced surveillance network. Promoted by country capacity-building visits from the WHO/IST to other meningitis belt countries, the number of reporting countries increased to 13 in 2004. The phased introduction of the group A meningococcal conjugate vaccine (PsA-TT) by mass campaigns across the meningitis belt since 2010 [6] further drove country participation in enhanced surveillance. By 2013, 19 countries (Benin, Burkina Faso, Cameroon, Central African Republic, Chad, Democratic Republic of Congo, Ethiopia, Ghana, Guinea, Côte d'Ivoire, Mali, Mauritania, Niger, Nigeria, Senegal, South Sudan, Sudan, The Gambia, and Togo) reported to this network. We describe the epidemiology of meningitis in countries that reported to the meningitis bulletins each year from 2004 to 2013, including the initial impact of PsA-TT on meningitis incidence.

## METHODS

### Definitions

#### Suspected Meningitis

A case of suspected meningitis was defined as any person with sudden onset of fever (>38.5°C rectal or 38.0°C axillary) and 1 of the following signs: neck stiffness, flaccid neck (infants), bulging fontanelle (infants), convulsion, or other meningeal signs [3].

#### Confirmed Meningitis

A case of confirmed meningitis was defined as any person with meningeal signs and isolation of a causal pathogen (*N. meningitidis*, *Streptococcus pneumoniae* [Spn], *Haemophilus influenzae* type b [Hib]) from the CSF by culture, PCR, or rapid diagnostic test [3].

#### Alert Threshold

The alert threshold was defined as an attack rate of 5 suspected cases per 100 000 inhabitants per week in a district or subdistrict (in populations ≥30 000); or as 2 cases in 1 week, or a higher incidence than in a nonepidemic year (in populations <30 000) [5]. Crossing this threshold triggers the reinforcement of surveillance.

#### Epidemic Threshold

The epidemic threshold was defined as an attack rate of 15 suspected cases per 100 000 inhabitants in 1 week in a district or subdistrict, or 10 per 100 000 if considered at high risk of an epidemic (in populations ≥30 000); or as 5 cases in 1 week or a doubling of incidence in a 3-week period (in populations <30 000) [7]. Crossing this threshold triggers the launch of vaccination campaigns when the predominance of *N. meningitidis* is confirmed and the use of a specific antibiotic treatment protocol.

### Data Analysis

Countries were included in this study if they contributed data to WHO/IST each year between 2004 and 2013 (Benin, Burkina Faso, Chad, Democratic Republic of Congo, Ghana, Ivory Coast, Mali, Niger, Nigeria, Togo). After de-duplication and reconciliation of data between the bulletins and the database, data were extracted and analyzed using Microsoft Excel 2010, version 10 (Microsoft Corporation). Data on numbers of cases and deaths were taken from the database, and data on pathogens and numbers of districts in alert and epidemic were taken from the bulletins at week 52 of each year. Where the database was incomplete—that is, all countries in 2004, Chad in 2005, and Nigeria in 2005 and 2006—all data were taken from the bulletins. Population estimates for 2004 to 2013 obtained from the United Nations Statistics Division [8] and the World Development Indicators were used to calculate overall incidence rates.

## RESULTS

**Figure 1.**
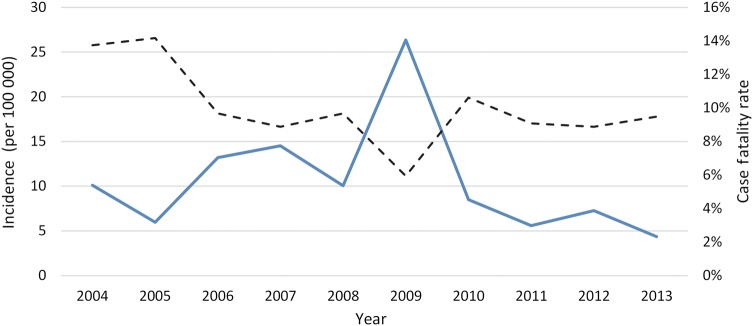
Incidence rate of suspected and confirmed cases of meningitis in the African meningitis belt by year (solid line) and case fatality (dotted line), 2004–2013.

**Figure 2.**
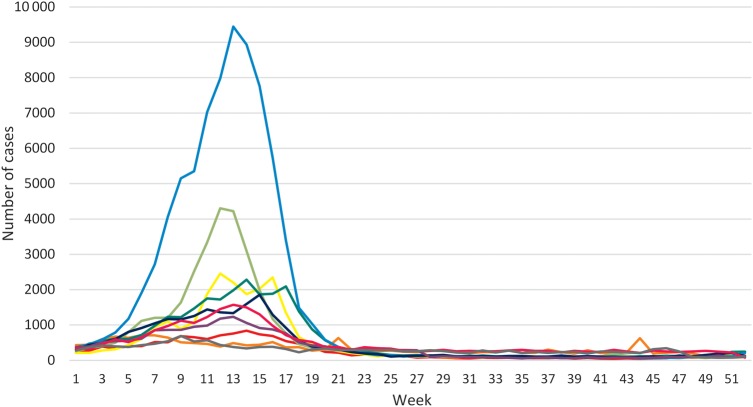
Number of suspected and confirmed meningitis cases by week in the African meningitis belt of 2004 (red), 2005 (orange), 2006 (yellow), 2007 (light green), 2008 (green), 2009 (light blue), 2010 (blue), 2011 (purple), 2012 (pink), and 2013 (gray).

A total of 341 562 suspected and confirmed cases were reported from the 10 countries, with considerable variation by country and year (Table 1). The irregular cyclical nature of meningitis epidemics is shown in Figures 1 and 2, with the largest number of cases in 2009 (n = 86 714) corresponding to a major epidemic in Nigeria and Niger, and the second-largest peak in 2007 (n = 45 195) due to an epidemic in Burkina Faso (Table 1). Case fatality was highest in 2004 and 2005 (13.7%) and remained close to 10% in subsequent years apart from 2009, the year of highest incidence, when fatality fell to 5.9%. The weekly number of meningitis cases by year (Figure 2) demonstrates the main seasonal peaks in the first 5 months of the year. Over the 10 years, 657 of 1470 reporting districts (on average, number of reporting districts varies widely over the years) in the 10 countries reached the alert threshold and 615 reached the epidemic threshold in 1 or more years. This corresponds to, respectively, an average across the 10-year span of 66 (range, 11–119) and 62 (range, 5–216) districts annually.

**Table 1.** Suspected and Confirmed Meningitis Cases in 10 Countries of the African Meningitis Belt, 2004–2013

Year	Benin	Burkina Faso	Chad	DRC	Ghana	Côte d'Ivoire	Mali	Niger	Nigeria	Togo	Total
2004	377	6252	863	13 367	982	464	1480	4081	659	305	28 830
2005	303	3626	1015	9082	430	527	454	1404	657	333	17 831
2006	316	19 134	1430	6109	371	705	1039	4465	5731	578	39 878
2007	502	26 878	1452	8645	461	760	977	1097	2642	723	44 137
2008	414	10 401	1093	8406	281	1131	1538	3757	6835	428	34 284
2009	416	4723	1505	9150	201	289	335	13 449	56 128	350	86 546
2010	323	6732	3147	8160	1129	151	482	2908	4983	460	28 475
2011	269	3875	5960	5167	773	141	430	1189	1165	440	19 409
2012	1165	6957	3874	10 142	956	500	688	314	1206	408	26 210
2013	833	2917	371	9326	454	255	358	311	871	266	15 962
Total	4918	91 495	20 710	87 554	6038	4923	7781	32 975	80 877	4291	341 562

Abbreviation: DRC, Democratic Republic of Congo.

From 2004 to 2013, 15 001 positive results from CSF samples were reported, of which 40% were positive for NmA, 26% for Spn, 18% for NmW, 8% for other types of *N. meningitidis*, 4% for Hib, and 3% for other pathogens. Niger and Burkina Faso contributed the highest numbers of laboratory-confirmed cases (45% and 28% of all confirmed cases, respectively). The proportion of suspected cases with an organism isolated rose from 3.9% in 2004–2010 to 6.9% in 2011–2013.

**Figure 3.**
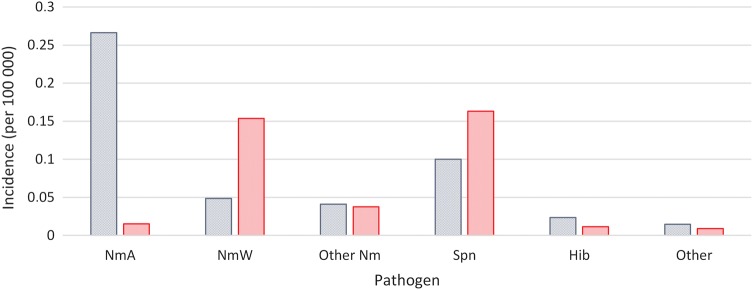
Incidence rate of pathogens per 100 000 population among positive cerebrospinal samples before (2004–2010) (blue) and after (2011–2013) (red) the introduction of vaccination with PsA-TT (MenAfriVac). Abbreviations: Hib, Haemophilus influenzae type b; Nm, *Neisseria meningitidis*; NmA, *Neisseria meningitidis* group A; NmW, *Neisseria meningitidis* group W; Spn, *Streptococcus pneumoniae*.

Table 2 and Figure 3 show the changing distribution of individual pathogens from confirmed cases of bacterial meningitis over the study period. NmA was the main cause of meningitis until the introduction of PsA-TT in 2010. NmW was detected more frequently between 2010 and 2013, and an epidemic of NmX was seen in Niger in 2006. Other types of *N. meningitidis* (NmB, NmC, and NmY) were uncommon: between 2010 and 2013, only 1 NmB case was confirmed in Côte d'Ivoire in 2011 and 2 NmB cases in Ghana in 2012. Spn remained an important cause of meningitis throughout the study period. Cases due to Hib were found more frequently in 2004–2005 than in subsequent years (11.1% of confirmed cases in 2004–2005 vs 3.2% in 2006–2013; χ^2^ with Yates correction, 236.0; *P* > .0001).

**Table 2.** Confirmed Meningitis Cases and Organisms Isolated From Cerebrospinal Fluid in Countries of the African Meningitis Belt, 2004–2013

Year	Total Confirmed Cases	NmA	NmW	NmC^a^	NmX^a^	Other Nm or Undetermined Serogroup^a^	Total Nm	Spn	Hib	Other
2004	1321	616	111			32	759	438	124	
2005	694	180	33			53	266	267	100	61
2006	1978	921	37		581	31	1570	258	83	67
2007	1086	609	62			9	680	282	74	50
2008	1449	1048	7			65	1120	242	48	39
2009	2590	1994	90			47	2131	350	34	75
2010	1633	430	718	4	55	13	1220	341	46	26
2011	1750	111	508	5	154	6	784	876	53	37
2012	1728	49	955	4	138	30	1176	487	40	25
2013	774	4	213	10	15	47	289	417	32	36
2004–2013	15 001	5962	2734	23	362	912	9993	3958	634	416

Abbreviations: Hib, *Haemophilus influenzae* type b; Nm, *Neisseria meningitidis*; NmA, *Neisseria meningitidis* group A; NmC, *Neisseria meningitidis* group C; NmW, *Neisseria meningitidis* group W; NmX, *Neisseria meningitidis* group X; Spn, *Streptococcus pneumoniae*.

^a^ For 2004–2009, “Other Nm” includes NmC, NmX apart from 2006 data for Niger.

Comparing the period before (2004–2010) with the period after (2011–2013) the introduction of PsA-TT, the incidence rate of NmA meningitis fell >10-fold, from 0.27 per 100 000 to 0.02 per 100 000 (*P* < .0001). The incidence of meningitis due to NmW rose after 2010, but not that due to other *N. meningitidis* serogroups. It should be noted that a marked increase in the incidence of NmW meningitis occurred in the 2010 meningitis season (Table 2), before the introduction of PsA-TT at the end of 2010.

## DISCUSSION

The acceptability of the enhanced surveillance system in the meningitis belt is shown by the continuing and increasing participation of the countries in contributing to the weekly meningitis bulletin and the central database at Ouagadougou. The bulletins provide a valuable and timely demonstration of the evolution of meningitis through the meningitis season, both of pathogens responsible and of districts crossing the alert and epidemic threshold [9]. This system gives a rapid and important overview of the changing trends in incidence and pathogen distribution and helps in planning response to epidemics, for example, providing information on outbreaks to assist the International Coordinating Group on Vaccine Provision for Epidemic Meningitis Control (ICG) in decisions on vaccine stocks and release. Between 2004 and 2013, 81 country vaccine requests, targeting 248 districts, were submitted to the ICG and approved from the 10 countries mentioned in this study, followed by reactive vaccination campaigns (Communication with ICG Secretariat Focal Point). Alongside the benefits from the regional overview, the enhanced surveillance system has driven reinforcement of surveillance in the participating countries including training and strengthening laboratory capacity [9].

Not surprisingly, there are limitations to the data completeness and quality. Variation in incidence between countries may partly reflect different sensitivities and specificities of national surveillance systems, whereas variation between years within countries is more likely to be a true reflection of the well-known irregular fluctuations in meningitis incidence across the meningitis belt [1]. Of the 10 countries, Burkina Faso had the highest numbers of reported cases and also the highest mean annual incidence rate at 59 suspected cases per 100 000 population. A comparison of incidence rate by country was not presented, mainly because in many countries only part of the country is at high risk of meningitis, whereas surveillance reporting covers the whole country. Laboratory confirmation capacity also differs by country. Thus, the pathogen distribution presented largely reflects the results of Burkina Faso and Niger, as together they have contributed to >70% of laboratory-confirmed cases. Variable completeness of reporting to the database was observed especially at district level by year and by country. Suspected cases are notified based on a definition that may be interpreted differently in countries according to local practice, and the percentage of confirmed cases was low, although increasing. Duplication of data entry for the same district and week were seen particularly in 2 countries (Democratic Republic of Congo and Ghana) where there was more uncertainty about the quality of the surveillance system and where population data were not always updated after changes in district boundaries. Data quality would be improved by database tools and electronic surveillance applications allowing capture and analysis of data in an electronic format at the lowest level possible for the respective country. Regular checks of data completeness and data quality, with regular feedback to the data-providing facilities, are also needed.

It is interesting that case fatality was highest in the first 2 years of this study, possibly due to subsequent improvements in case management and/or to changes in pathogen distribution. These 2 years saw the highest proportion of confirmed cases due to Hib, and an overall decline in reported Hib meningitis, likely associated with the rollout of Hib vaccines into childhood immunization schedules in all 10 countries between 2002 (Ghana) and 2012 (Nigeria). Such a decline is not observed with pneumococcal meningitis; pneumococcal vaccination was only introduced in 2011 in Benin and Mali, then in 2012 in Ghana, and in 2013 in Burkina Faso. The low case fatality in 2009 may reflect the high proportion of cases due to NmA or to the overall lower reported case fatality in the main countries affected that year (Nigeria and Niger; data not shown). In a systematic review of bacterial meningitis among African children, median case fatality for meningococcal meningitis was relatively low at 4% (range, 3%–6%) vs 25% (range, 4%–41%) for Hib [10]. Although fatality was highest for pneumococcal meningitis at 35% (range, 9%–67%), it is encouraging that the overall case fatality in this study did not rise after 2010 despite a higher proportion of confirmed cases due to Spn.

The weekly incidence data show the expected seasonal peaking of meningitis in the first 5 months of the year, although epidemics may start in December before the main season, and a small peak in incidence is noted in week 44 of 2005 (Figure 2). Although the risk factors predisposing populations to meningococcal outbreaks are poorly understood, the dry season with low absolute humidity and dust linked to the Harmattan winds are well known to be linked to this seasonal pattern [11]. Rising humidity and the onset of the rains are established predictors of the end of the epidemics [12].

Until 2010, NmA was the main cause of meningitis epidemics in Africa [6]. Since the introduction of mass population vaccination with the group A meningococcal conjugate vaccine, starting with Burkina Faso and parts of Mali and Niger at the end of 2010, and continuing until 2016 [13], the proportion of confirmed meningitis cases due to this serogroup fell dramatically, as recorded in Burkina Faso and Chad [14–16]. The incidence of NmW rose in the meningitis season of 2010 before the introduction of PsA-TT and has remained at higher levels since. An outbreak of NmX was seen in Niger in 2006 [17], and continues to cause a small proportion of cases, while an outbreak of NmC was recorded in northern Nigeria in 2013 and 2014 [18]. Other serogroups such as NmB and NmY are rarely isolated in the meningitis belt. As the rollout of PsA-TT campaigns was progressive between 2010 and 2013, the comparison before and after 2010 would benefit from more refined analyses; a mathematical model that takes account of the year of introduction of PsA-TT would help to more accurately document the impact of PsA-TT on NmA and other organisms. Although the rising proportion of laboratory confirmation among suspected cases is encouraging, continued improvement in surveillance, including the development and expansion of case-based surveillance [13], is needed to better detect any changes in serogroup and pathogen distribution and document the impact and long-term effectiveness of PsA-TT.
